# Dynamics of post-ejaculated intrauterine environment in mice

**DOI:** 10.17912/micropub.biology.001872

**Published:** 2025-11-12

**Authors:** Yu Matsumoto, Ban Sato, Masafumi Inui, Natsuko Kawano, Kenji Miyado

**Affiliations:** 1 Laboratory of Regulatory Biology, Department of Life Sciences, School of Agriculture, Meiji University; 2 Laboratory of Animal Regeneration Systemology, Department of Life Sciences, School of Agriculture, Meiji University; 3 Department of Reproductive Biology, National Research Institute for Child Health and Development; 4 Division of Diversity Research, National Research Institute for Child Health and Development

## Abstract

After ejaculation, the intrauterine environment undergoes dynamic fluid changes due to post-ejaculated uterine fluid (eUF) coagulation and subsequent liquefaction. These changes presumably contribute to fertilization and reproductive efficiency; however, their physiological roles remain unclear. We studied the significance of the post-ejaculated intrauterine environment during in vivo fertilization. eUF coagulated immediately after ejaculation, and histological analysis of the uterus suggested that eUF liquefaction was promoted 6–10 h post-ejaculation. However, most gametes completed fertilization within 4 h post-ejaculation. Since eUF fluid changes did not align with fertilization timing, they are assumed to contribute to reproductive phenomena beyond sperm transport and release.

**Figure 1. Post-ejaculated intrauterine environment in mice:**
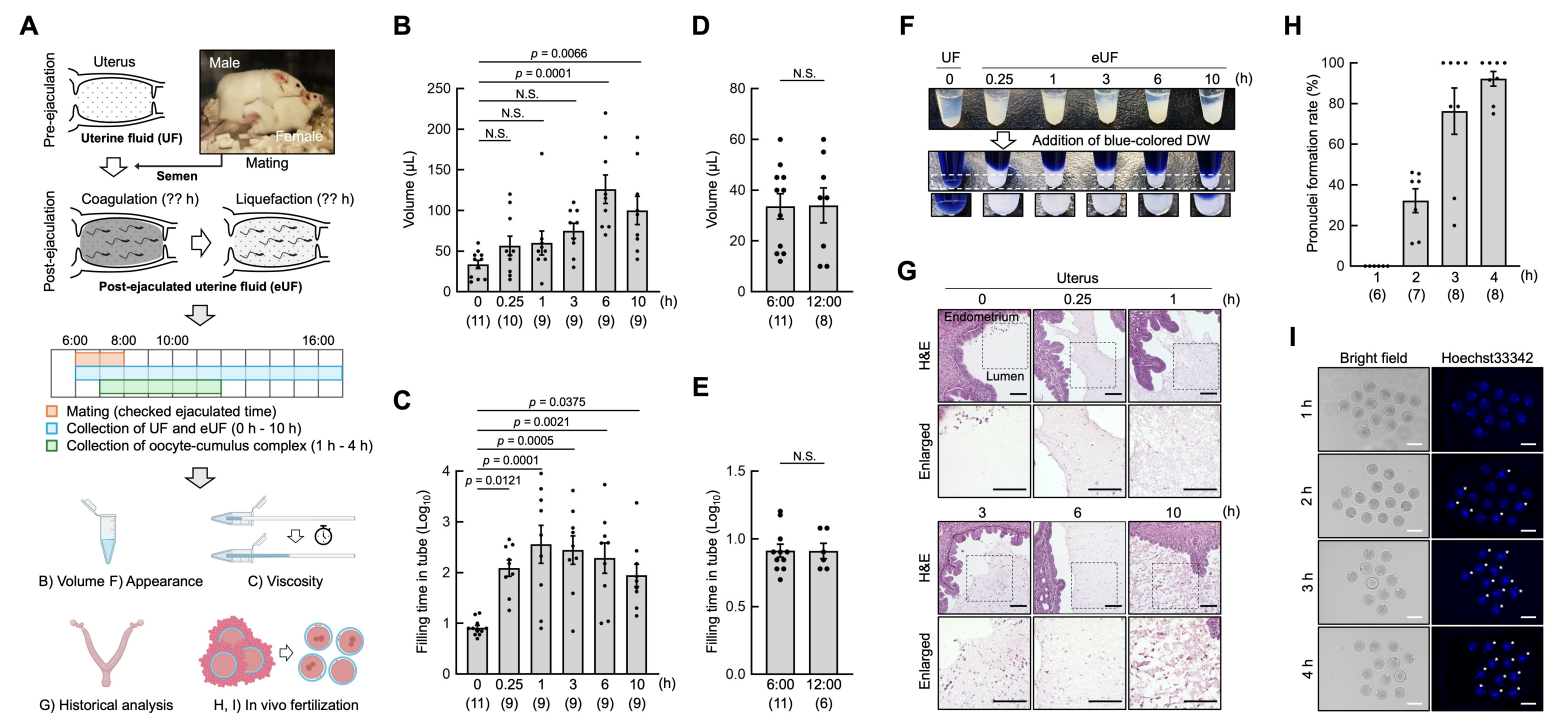
(A) Experimental flow. Part of this figure was created with BioRender.com. (B, C) Volume (μL) and viscosity of UF (0 h) and eUF (0.25–10 h) collected from the uterus pre- and post-ejaculation. Viscosity was assessed based on the time taken for the collected UF and eUF to fill a 20 µL capillary tube (seconds = Log10). N.S., not significant. (D, E) Volume (μL) and viscosity of UF collected at 6:00 (mating time) and 12:00 (equivalent time to 6 h post-ejaculation). (F) Appearance of UF and eUF. upper image; after centrifuged (5,000 × *g* , 5 min); middle image, after added bule-colored DW; dotted box, image enlarged below; white precipitates, coagulation; blue-colored DW, 0.015% Trypan blue solution. (G) Histological analysis of the pre- and post-ejaculated uterus using H&E staining. Bottom panels are enlarged images of dotted boxes in top panels. Scale bars = 100 μm. (H, I) In vivo fertilization rate of eggs collected from the ampulla of the oviduct post-ejaculation (1–4 h). Eggs were developed in KSOM until 16:00, and were stained with Hoechst 33342 (blue). White asterisk, pronuclei; scale bars = 100 μm.

## Description

Mammals reproduce by internal fertilization, whereby the male ejaculates semen into the uterus or vagina. In mice, semen is ejaculated into the uterus, and the post-ejaculated intrauterine environment is filled with post-ejaculated uterine fluid (eUF), a mixture of uterine fluid (UF) and semen ( Figure 1A ). The volume of eUF increases ( Figure 1B ) in this intrauterine environment, and the fluid changes similarly to that observed in human semen, where eUF changes into a high-viscosity fluid (coagulation) and then into a low-viscosity fluid (liquefaction) (Miki & Clapham 2013; Li et al. 2017). Ejaculated human semen coagulates due to semenogelin (SEMG) and fibronectin derived from the seminal vesicle fluid (Lilja et al. 1987), and liquefies as SEMG is broken down by the protease activity of prostate-specific antigen (PSA) derived from the prostatic fluid (Lilja 1985; Robert et al. 1997). This liquefaction reaction is induced within 20 min post-ejaculation, enabling the sperm to migrate within the female reproductive tract. Therefore, disturbances such as hyperviscosity and liquefaction defects in human semen are hypothesized as a contributor to male infertility (Lin et al. 1992; Esfandiari et al. 2008). However, the time course of coagulation and liquefaction induced by eUF and their effects on reproductive processes in mice remain unclear.

To clarify these issues, we have collected UF and eUF and studied the series of reproductive phenomena involving coagulation, liquefaction, and its relationship between fertilization ( Figure 1A ). The viscosity of eUF increased immediately after semen deposition in the uterus (0 h, 0.91 ± 0.05 vs 0.25 h, 2.09 ± 0.16; *p* = 0.0121), was the highest at 1 h post-ejaculation (2.56 ± 0.37; *p* = 0.0001), and decreased over time thereafter. In contrast, the volume and viscosity of the UF remain constant during estrus without ejaculation. This result indicates that the increase in the volume and viscosity of eUF is a specific phenomenon observed in the post-ejaculated intrauterine environment. When the appearance of UF and eUF was examined, white coagulated precipitates with increased viscosity were observed in eUF but not in UF ( Figure 1F ). Consistent with the results shown in Figure 1F, histological analysis of the uterine components before and after ejaculation using hematoxylin and eosin (H&E) staining showed coagulated contents only in the post-ejaculated uterine lumen ( Figure 1G ). Focusing on the uterine lumen at 10 h post-ejaculation, the coagulated contents were dispersed, suggesting that this phenomenon may be related to liquefaction. However, when the time of fertilization within the female reproductive tract after ejaculation was examined, most eggs were fertilized with sperm within 3–4 h post-ejaculation (Figures 1H and 1I). Previous studies have reported that ejaculated sperm pass through the uterotubal junction and reach the isthmus of the oviduct within 1.5 h post-ejaculation (La Spina et al. 2016), and sperm that contact an egg in the ampulla of the oviduct penetrate the zona pellucida within approximately 20 min (Sato & Blandau 1979). Therefore, it is unlikely that sperm migrate to the oviduct due to the liquefaction of eUF in mice. These results suggest that coagulation and liquefaction of eUF in mice and coagulation and liquefaction of semen in humans have distinct physiological significances.

We demonstrated a temporal relationship between fluid changes in the post-ejaculated intrauterine environment and fertilization. Although the molecular mechanisms and physiological significance of coagulation and liquefaction in eUF during in vivo fertilization remain unclear, our results contribute to elucidating the entire process of in vivo fertilization within the female reproductive tract.

## Methods

**Animals**

Female and male ICR mice (8–12-week-old) were purchased from Japan SLC, Inc. (Shizuoka, Japan).

All experiments were performed with the approval of the Animal Care Committee of Meiji University (approval numbers: IACUC15−0014, MUIACUC2020−04, and MUIACUC2025−08). The mice were housed under standardized light-dark cycle conditions (lights on at 5:00 and off at 19:00). Food and water were provided ad libitum.

**Volume, viscosity, and appearance of UF and eUF**

UF and eUF were collected from the uterus of 8–12-week-old female ICR mice. Mating of 8–12-week-old male ICR mice was performed between 6:00 and 8:00. UF was collected from estrous female mice that allowed for male mounting behavior (UF = 0 h post-ejaculation), and eUF was collected from estrous female mice that allowed for male mounting behavior and confirmed ejaculation. Ejaculation was confirmed by the male’s temporary rigidity while holding the female after mounting behavior with anteroposterior movement of the loin. After sacrifice females, a P-200 pipette with a 200 μL tip was inserted into the cervix to collect UF and eUF in uterus. The volume was measured using a 20 µL capillary tube (Drummond Scientific Company, Broomall, PA, USA), and the viscosity was assessed by measuring the time to fill a 20 µL capillary tube, as described previously (Rijnders et al. 2007). For UF <20 µL, the time to fill 10 µL was measured, and the time to fill 20 µL was predicted by doubling the measured time. After centrifuging (5,000 × *g* , 5 min) 30 µL of UF and eUF, 30 µL of 0.015% Trypan blue solution (Nacalai Tesque Inc., Kyoto, Japan) was added to visualize the coagulated precipitates.

**Histological analysis of the pre- and post-ejaculated uterus**

The uterus was fixed with Bouin’s solution (FUJIFILM Wako Pure Chemical Corp., Osaka, Japan) and embedded in optimal cutting temperature compound (Sakura Finetek Japan Co., Ltd., Tokyo, Japan) after replacement with 30% (v/w) sucrose (FUJIFILM Wako Pure Chemical). H&E staining of 5 μm sections was performed using standard techniques. Briefly, the sections were stained with hematoxylin solution (New Hematoxylin Solution Type M; Muto Pure Chemicals Co., Ltd., Tokyo, JAPAN) for 5 min and eosin solution (1% Eosin Y solution; Muto Pure Chemicals) for 2 min. After staining, the sections were dehydrated with ethanol (Muto Pure Chemicals) and xylene (Muto Pure Chemicals), and enclosed with New M・X (Matsunami Glass Industry Co., Ltd., Osaka, Japan).

**Evaluation of the timing of in vivo fertilization**

The oocyte-cumulus complex (OCC) was collected from the ampulla of the oviduct after ejaculation (1, 2, 3, and 4 h). The OCCs were collected in potassium simplex optimized medium (KSOM; Sigma-Aldrich Co. LLC., St. Louis, MO, USA) and washed once with fresh KSOM. At 16:00 on the same day, the cumulus cells were removed from the OCC by incubation with 1 mg/mL hyaluronidase (Sigma-Aldrich) for 10 min at room temperature (25–27 ℃), and the eggs were collected. The eggs were stained with Hoechst 33342 (1:500, DOJINDO Laboratories, Kumamoto, Japan) by incubation at room temperature for 5 min. Eggs were visualized using a BZ-X700 fluorescence microscope (Keyence Corp., Osaka, Japan), and the in vivo fertilization rate was assessed by counting the number of eggs that formed pronuclei.

**Statistical analysis**

Values of results are expressed as means ± standard error of the mean (SEM). The numbers of mice examined are indicated in parentheses. The probability of statistical significance was calculated using one-way analysis of variance with Tukey’s multiple comparison test (Figures 1B and 1C) and Student’s *t* -test (Figures 1D and 1E).
